# Laser Peripheral Iridotomy versus Trabeculectomy as an Initial Treatment for Primary Angle-Closure Glaucoma

**DOI:** 10.1155/2017/2761301

**Published:** 2017-09-01

**Authors:** Yan Yun Chen, Su Jie Fan, Yuan Bo Liang, Shi Song Rong, Hai Lin Meng, Xing Wang, Ravi Thomas, Ning Li Wang

**Affiliations:** ^1^Beijing Tongren Eye Center, Beijing Tongren Hospital, Capital Medical University, Beijing Key Laboratory of Ophthalmology and Visual Sciences, Beijing, China; ^2^Handan Eye Hospital, Handan, Hebei Province, China; ^3^The Affiliated Eye Hospital, School of Optometry and Ophthalmology, Wenzhou Medical University, China; ^4^School of Medicine, Dentistry and Biomedical Sciences Public Health, Health Services and Primary Care, Queen's University, Belfast, UK; ^5^Department of Ophthalmology, Massachusetts Eye and Ear, Harvard Medical School, Boston, MA, USA; ^6^Anyang Eye Hospital, Anyang, Henan Province, China; ^7^Fushun Eye Hospital, Fushun, Liaoning Province, China; ^8^Queensland Eye Institute, Brisbane, Queensland, Australia; ^9^University of Queensland, Brisbane, Queensland, Australia; ^10^Beijing Institute of Ophthalmology, Beijing Tongren Eye Center, Beijing Tongren Hospital, Capital Medical University, Beijing Key Laboratory of Ophthalmology and Visual Sciences, Beijing, China

## Abstract

**Purpose:**

To compare laser peripheral iridotomy (LPI) with trabeculectomy as an initial treatment for primary angle-closure glaucoma (PACG) with peripheral anterior synechiae (PAS) ≥ 6 clock hours.

**Methods:**

Patients were drawn from two randomized controlled trials. 38 eyes of 38 patients (PAS ≥ 6 clock hours) were treated with LPI (group 1) while 111 eyes of 111 PACG patients (PAS ≥ 6 clock hours) underwent primary trabeculectomy (group 2). All patients underwent a comprehensive ophthalmic examination at baseline and at postoperative visits and were followed up for a minimum of one year.

**Results:**

Group 2 had higher baseline IOP (45.7 ± 14.8 mmHg versus 34.3 ± 14.3 mmHg) than group 1 and more clock hours of PAS (10.4 ± 1.9 versus 9.0 ± 2.2). IOPs at all postoperative visits were significantly lower in group 2 than in group 1 (*p* = 0.000). Five eyes in group 1 required trabeculectomy. 17 of the 38 eyes in group 1 (44.7%) required IOP-lowering medications as compared to seven of the 111 eyes in group 2 (6.3%). Cataract progression was documented in 2 eyes (5.3%) in group 1 and 16 eyes (14.4%) in group 2.

**Conclusions:**

Primary trabeculectomy for PACG (PAS ≥ 6 clock hours) is more effective than LPI in lowering IOP.

## 1. Introduction

Primary angle-closure glaucoma (PACG) is the major type of primary glaucoma in China [1–4]. The management of PACG in China is different from the recommendations of the American Academy of Ophthalmology Preferred Practice Patterns (PPP) and other published guidelines [5–7]. For primary angle closure (PAC) and PACG, the PPP recommends laser peripheral iridotomy (LPI) to eliminate pupillary block followed by a treatment strategy “similar to that for POAG” [8]. Accordingly, LPI is used as the first-line treatment for all patients with PAC or PACG, medication is added as needed, and surgery is considered when the intraocular pressure (IOP) cannot be controlled with maximum tolerated medications. In China, however, trabeculectomy is considered a primary option for PACG and is generally undertaken if peripheral anterior synechiae (PAS) are greater than 6 clock hours [9, 10]. To the best of our knowledge, there is no comparative study of LPI versus primary trabeculectomy in PACG to support such an approach. Using data from patients enrolled in two separate randomized controlled trials [11, 12], we compared the IOP-lowering efficacy and safety of LPI versus trabeculectomy as an initial treatment for PACG with PAS ≥ 6 clock hours.

## 2. Methods

Patients included in this study had participated in 2 randomized clinical trials (RCTs) for PACG. Both trials were conducted in accordance with the tenets of the Declaration of Helsinki and approved by the ethics committee of the Tongren Eye Centre, Capital Medical University. Written informed consent was obtained from all subjects for participation in the original trials.

Patients undergoing primary trabeculectomy for PACG had participated in a multicenter RCT (registration number: ChiCTR-TCR-00000218) [12]. This RCT was conducted in four clinical collaborative centers of Beijing Tongren Hospital: Handan 3rd Hospital (Hebei Province, China), Anyang Eye Hospital (Henan Province, China), Fushun Eye Hospital (Liaoning Province, China), and the Chenzhou Eye and Optometry Center (Hunan Province, China). PACG was defined as primary angle closure with glaucomatous optic neuropathy and corresponding visual field defects, and patients were recruited from the four centers between April 2006 and November 2007. The primary purpose of the trial was to report the efficacy and complications of trabeculectomy with or without releasable sutures in PACG.

Patients who underwent LPI as an initial treatment were part of another RCT (registration number: ChiCTR-TRC-00000034, http://www.chictr.org.cn) conducted at the Handan 3rd Hospital [11]. The purpose of this RCT was to investigate the role of laser iridotomy (with or without iridoplasty) in patients with synechial PAC or PACG. The definition of PACG was the same as that used in the trial mentioned above; consecutive cases of PAC and PACG presenting to the hospital between October 1, 2005, and October 31, 2006, were recruited for this trial.

### 2.1. Patient Selection

The inclusion criteria for the current study were as follows:

1. PACG: defined as primary angle closure with glaucomatous optic neuropathy and visual field defects [13]
2. Age 40 years or more
3. PAS ≥ 6 clock hours
4. Minimum follow-up of one year.

Patients with acute angle-closure glaucoma were excluded.

As part of the original clinical trial, all patients had undergone a comprehensive ophthalmic examination including refraction, Goldmann applanation tonometry, static and dynamic gonioscopy (manipulation) using a one-mirror Goldmann gonioscope [14, 15], slit-lamp examination, fundus examination, and automated perimetry (Humphrey Field Analyzer 750i, Carl Zeiss Meditec; Sita Fast strategy; and 24–2 threshold test). These examinations were performed at baseline and at postoperative visits scheduled at month 1, month 3, month 6, month 12, and month 18. Postoperative visits were scheduled on day 1, day 3, week 1, week 2, month 1, month 3, month 6, month 12, and month 18 following the LPI or trabeculectomy.

### 2.2. Laser Peripheral Iridotomy

All laser procedures were performed by one of two senior glaucoma specialists. 2% pilocarpine was applied, and iridotomy was performed under topical anesthesia using a Nd:YAG laser (YL-1600; NIDEK Co. Ltd., Japan) using an Abraham contact lens (Ocular Instruments Inc., Bellevue, USA). A treatment site was selected in the superior nasal iris or in a crypt where present. The treatment was initiated with a single 4 mJ pulse, the power was adjusted, and the treatment was continued to obtain a 0.2 mm opening; patency was determined by direct visualization of the posterior chamber.

In accordance with local practice, IOP-lowering medication was initiated if the IOP was greater than 21 mmHg following laser and confirmed by a repeat reading on the same day [11].

### 2.3. Trabeculectomy

Surgery was performed under topical or peribulbar anesthesia using a standard surgical technique. The eye was prepared using a standard aseptic technique and draped to isolate the lashes. A lid speculum was inserted and a 7/0 superior rectus muscle traction suture was placed. A limbus-based conjunctival flap was created using a 10 mm incision through the conjunctiva and Tenon's capsule approximately 8–10 mm from the limbus. The flap was dissected forwards and hemostasis achieved with monopolar diathermy.

A half-thickness 4 × 3 mm^2^ rectangular scleral flap was fashioned, and cellulose sponges soaked in MMC (0.3 mg/ml) were applied under the scleral flap, conjunctiva, and Tenon's capsule for a duration determined by the surgeon based on an assessment of risk factors. Irrigation with balanced salt solution was performed to wash out residual MMC solution. A paracentesis was created, a 2 × 1.5 mm trabeculectomy block excised, and an iridectomy performed. The scleral flap was sutured with 10-0 monofilament, BSS was injected into the anterior chamber to assess flow, and the conjunctiva was closed with a single running 8/0 vicryl suture [12].

Visual acuity was recorded with a decimal chart and converted to the logarithm of minimum angle of resolution (LogMAR) format. Finger counting, hand movement, and light perception were recorded as 1.5, 2.0, and 2.5 on the LogMAR scale.

At each postoperative visit, a trained technician measured the IOP twice using an applanation tonometer and recorded the average. If the difference between the two measurements was more than 2 mmHg, a third measurement was performed, and the average of the two closer results was recorded. All IOP measurements were performed between 8 am and 12 am.

At each site, trained observers used standard LOCS III photographs to assess the lens. Cataract progression was defined as an increase of 2 or more units greater than baseline in any LOCS III category (nuclear, cortical, or posterior capsular opacity). Progression was analyzed in all eyes with a minimum follow-up of 12 months; for those whose longer follow-up was available, it was determined at the 18-month visit. The shallow anterior chamber (AC) was categorized as grade I = peripheral iris-cornea touch; grade II = midiris-cornea touch; and grade III = central cornea-lens touch [16]. Hypotony was defined as IOP ≤ 5 mmHg [17].

At each visit, other complications and interventions, if undertaken, were recorded.

### 2.4. Statistical Analysis

One eye of each patient was randomly selected for the analysis.

Statistical analysis was carried out using SPSS 15.0 (SPSS Inc., Chicago, USA). Independent *t*-test was used to compare the difference between the groups, and the chi-square test was used to compare the difference in IOP-lowing medication and cataract progression between the groups. A *p* value < 0.05 was considered significant.

## 3. Results

38 eyes of the 38 patients with PACG that underwent primary iridotomy comprised group 1 while group 2 consisted of 111 eyes of the 111 PACG patients who had undergone trabeculectomy.

The baseline characteristics of the two groups are summarized in Table 1. Patients in group 2 were slightly younger and had higher baseline IOP and more clock hours of PAS.

Table 2 shows the postoperative IOP and visual acuity. At each visit, the IOP was significantly lower in group 2 (*p* < 0.001).

Five eyes in group 1 required trabeculectomy: 1 eye at 6 months, 2 eyes at 12 months, and 2 eyes at 18 months. 17 of the 38 eyes (44.7%, CI: 28.9%–60.5%) in group 1 required a mean of 1.8 IOP-lowering medications while seven of the 111 eyes (6.3%, CI: 1.8%–10.8%) in group 2 required a mean of 1.1 medications (Pearson's chi-square value = 30.940, *p* < 0.001).

19 eyes in group 2 developed a transient shallow anterior chamber (AC), but there was no instance of lens-cornea touch. 16 eyes (including 13 eyes with shallow AC) experienced transient hypotony. Hypotony lasted one day in 14 eyes and one week in 2 eyes and all recovered spontaneously. One eye developed hypotony maculopathy at one month after surgery that was resolved after IOP increased 3 weeks later. In one eye, the IOP increased to 60 mmHg at 3 months postsurgery and required cyclophotocoagulation.

Best-corrected visual acuity at 18 months was 0.5 ± 0.5 in group 1 and 0.6 ± 0.7 in group 2. Four eyes (10.5%) in group 1 lost one line of vision, 3 eyes (7.9%) lost 2 lines, and 3 (7.9%) lost more than 2 lines. In group 2, thirteen eyes (11.7%) lost 1 line, 9 eyes (8.1%) lost 2 lines, and 11 eyes (9.9%) lost more than 2 lines. Six eyes in group 1 and 20 eyes in group 2 lost ≥2 lines (Pearson's chi-square value 0.098, *p* = 0.755). The absolute risk for ≥2 lost lines of vision in the trabeculectomy group was 18% (20 of 111) compared to 16% (6 of 38) in the LPI group. The absolute risk increased with trabeculectomy is 2% and the number needed to harm (NNH) is 50.

Cataract progression as defined was documented in 2 eyes (5.3%, CI: −1.8%–12.4%) in group 1 and 16 eyes (14.4%, CI: 7.9%–20.9%) in group 2 (Pearson's chi-square value = 2.232, *p* = 0.135). The absolute risk increased in cataract formation with trabeculectomy is 9% and translates into a NNH of 11.

## 4. Discussion

The PACG treatment strategy formulated by the glaucoma group of the Chinese Ophthalmology Society is different from that formulated by other published guidelines. As per Chinese guidelines, LPI is suggested for “patients with PAS < 180° without optic disc and visual field damage” (an indication endorsed without data by a recent publication), while primary trabeculectomy is recommended for PACG with PAS ≥ 180° [9, 18]. A 2005 survey of clinical practice in PACG diagnosis and treatment found that 73% of Chinese glaucoma specialists preferred trabeculectomy as the initial treatment for PACG with PAS ≥ 180° [10]. Evidence for such an approach is however lacking, and to the best of our knowledge, our study is the first study to compare primary LPI with primary trabeculectomy for PACG.

LPI is considered a noninvasive, simple, and safe intervention in PACS, PAC, and PACG [19]. The effect of a LPI would depend on the amount of angle that is available for filtration, and this can be estimated clinically by the extent of PAS [18, 20]. IOP control following LPI as reported in the literature varies considerably, and the therapeutic effect seems to decrease over time [21, 22]. In a retrospective analysis of 131 cases (251 eyes) in China, 18 eyes with PACS, 98 eyes with PAC, and 129 eyes with PACG that underwent LPI were followed up for 9.2 ± 3.7 years [23]. Eyes with PAC and PACG had PAS < 6 clock hours. At the final follow up, 16/18 PACS, 38/98 PAC, and 814/129 of PACG eyes were controlled (defined as IOP less than 21 mmHg) without medications. 60% of cases required additional medications and 13% needed filtering surgery.

A study from India evaluated the long-term outcome of PACG following laser iridotomy [24]. 70 consecutive patients with PACG whose IOP remained >21 mmHg despite a patent iridotomy had their IOP controlled by medications or trabeculectomy and were followed up over a 6-year period. A trabeculectomy without antimetabolites was performed if IOP was >21 mmHg on maximal tolerable medical therapy, if there was evidence of progression of the disc/visual field, or if the patient was noncompliant. 46 (65.7%) eyes were controlled medically with 26 (57%) eyes requiring two topical medications. 24 (34.3%) eyes required trabeculectomy at various times during follow-up. The extent of PAS was not reported in this study, but reports suggest that the therapeutic effect of LPI decreases over time and many cases need filtering surgery.

The results seem consistent with the biologically plausible hypothesis that LPI is more successful in those with relatively undamaged trabecular meshwork [18]. The literature seems to suggest that, despite the presence of a patent LPI, eyes with established PACG and a certain (currently undefined) extent of PAS require further treatment to control IOP and that medical therapy fails in a significant number of cases necessitating filtering surgery [18, 25].

In the current investigation, the IOP during follow-up was significantly lower in the trabeculectomy group. Furthermore, a larger number of patients who underwent LPI needed medical treatment as compared to those who underwent trabeculectomy. In this developing country, setting patients may not be able to afford medical treatment or are generally not adherent or persistent with it. To simulate a real-world condition, we did not provide free IOP-lowering medication and found that 24% of patients could not afford long-term medication. Moreover, 5 eyes (13.2%) of patients who underwent LPI with baseline PAS ≥ 6 clock hours (group 1) had to undergo trabeculectomy during the follow-up period.

The poor effectiveness of IOP lowering following LPI for PACG with ≥6 hours of PAS would seem important in selecting treatment within the well-known constraints of a developing country, including a possible one shot at treatment. We therefore suggest that 6 clock hours of PAS can be considered a provisional clinical threshold for LPI in PACG, as this is likely to change with further research and will be different for individual cases. Our findings lend credence to a recently published algorithm for the management of angle-closure disease that recommended a ≥6 clock hour threshold for PAS; that algorithm was based mainly on biological plausibility [18]. Considering the problems of medical treatment in developing countries, a policy of primary trabeculectomy for PACG with ≥6 clock hours of PAS might merit consideration. In fact, even eyes with <6 clock hours of PAS may be considered for a trabeculectomy if the socioeconomic situation so dictates [23].

The choice of initial treatment must also consider the issue of complications. Consistent with most other publications, we did not encounter any significant complications in the LPI group [11]. While complications with trabeculectomy are more frequent, the incidence of severe sight-threatening complications is now less than what it was 20 years ago; we encountered mainly transient shallow chamber and hypotony. It must be kept in mind that in our study, trabeculectomy was undertaken by glaucoma specialists in the context of a clinical trial and the potential for sight-threatening complications remains. Importantly, even in the context of a clinical trial, 18% of eyes undergoing trabeculectomy lost 2 or more lines of vision. However, the number needed to harm for ≥2 lost lines of vision is an acceptable 50. Incision-induced astigmatism as well as progression of cataract was the major reason for visual loss, but both would be considered treatable. 19% of patients in undergoing trabeculectomy had astigmatism of >1D at 6-month visit, and 14.4% had obvious progression of lens opacity. The NNH for progression of cataract with trabeculectomy was 11. While successful intervention is available for cataracts, this low NNH that could lead to an increase in the burden of cataract is a disadvantage with trabeculectomy.

The choice for the initial intervention for PACG in China and other developing countries must weigh up effectiveness versus complications as well as the need for additional medications and further interventions. While LPI is safe and easy, there is a more need for intensive medications and surgical intervention. The role of cataract extraction in primary angle-closure disease is evolving but is again more likely to be useful for those with angles less compromised by PAS [18, 26]. Trabeculectomy is better than LPI for lowering the IOP, but it does have the potential for more loss of visual acuity and for serious complications. Accordingly, it is probably best to reserve primary surgical intervention for established PACG with ≥6 clock hours as these are unlikely to do well with laser alone. While not statistically significant in this study, the higher chances for progression of cataract following trabeculectomy raise the question of combining filtration with cataract surgery, an issue not addressed in this study. In addition to clinical factors, any decision will also depend on the availability of lasers and surgeons and costs.

One of the limitations in our study is the small sample size in group 1 and the relatively short follow-up time. As few patients progressed during this time, we could not analyze the relationship between IOP and the visual field progression, but IOP would usually be considered an acceptable surrogate. Also, our results are derived from patients enrolled in RCTs, and the effectiveness in the real world is likely to be different. Furthermore, as it was not part of this study, we cannot comment on the increasingly popular role of phacoemulsification or its combination with trabeculectomy in the management of PACG [18]. The low NNH for cataracts with trabeculectomy could however be used as an argument for combining the two surgical procedures.

Finally, while the definition of PACG used in both trials was the same, patients for this study were drawn from two separate trials that asked different questions. This could introduce significant bias. The original LPI RCT (registration number: ChiCTR-TRC-00000034, http://www.chictr.org.cn) [11] that randomized patients into two different treatment groups (iridotomy or iridotomy plus iridoplasty) found no significant difference in IOP, medications, need for surgery, or visual function between groups at the 1-year visit. This RCT showed that in eyes with PACG, both iridotomy alone and iridotomy combined with iridoplasty provide a significant and similar reduction in IOP. For the trabeculectomy RCT (registration number: ChiCTR-TCR-00000218) [12], patients were randomly allocated to permanent or releasable sutures. This study too did not demonstrate any significant differences between the groups. Accordingly, we felt that analyzing patients from either arm of the two trials for the current study was acceptable but acknowledge that the possibility of unknown differences does exist and could affect interpretation. However, as the baseline IOP was higher and PAS more extensive in the trabeculectomy group, the conclusion in favor of trabeculectomy is likely to stand.

In conclusion, our results support the Chinese guidelines for management of PACG. In PACG with ≥6 clock hours of PAS, primary trabeculectomy is more effective than LPI in lowering IOP and significantly requires less medication but with more progression of cataract and more loss visual acuity. In developing countries, such as China, trabeculectomy can be considered a primary option for PACG with this degree of PAS.

## Figures and Tables

**Table 1:** Demographics.

Characteristics	Group 1	Group 2	*t* value	*p* value
Age	65.9 ± 7.9	62.3 ± 7.1	2.63	0.009
Baseline BCVA (LogMAR)	0.5 ± 0.5	0.5 ± 0.6	−0.033	0.974
Baseline IOP	34.3 ± 14.3	45.7 ± 14.8	−4.119	0.000
Baseline VCDR	0.7 ± 0.2	0.8 ± 0.1	−2.004	0.051
Baseline PAS	9.0 ± 2.2	10.4 ± 1.9	−3.892	0.000
Baseline MD	−17.3 ± 11.9	−19.2 ± 10.7	0.927	0.356

LPI = laser peripheral iridotomy; BCVA = best-corrected visual acuity; LogMAR = logarithm of minimum angle of resolution; IOP = intraocular pressure; VCDR = vertical cup/disc ratio; PAS = peripheral anterior synechiae; MD = mean deviation.

**Table 2:** IOP and final BCVA after LPI and trabeculectomy.

Characteristics	Group 1	Group 2	*t* value	*p* value
IOP 1 M	23.1 ± 10.0	14.0 ± 4.5	5.397	0.000
IOP 3 M	20.2 ± 6.8	13.1 ± 4.1	6.066	0.000
IOP 6 M	22.1 ± 6.7	13.6 ± 4.3	8.826	0.000
IOP 12 M	19.1 ± 5.3	14.5 ± 3.5	6.145	0.000
IOP 18 M	19.0 ± 5.3	14.9 ± 3.8	5.208	0.000
Final BCVA	0.5 ± 0.5	0.6 ± 0.7	−0.948	0.346

M = months; BCVA = best-corrected visual acuity; PAS = peripheral anterior synechiae; IOP = intraocular pressure.
